# How the known reference weakens the visual oblique effect: a Bayesian account of cognitive improvement by cue influence

**DOI:** 10.1038/s41598-020-76911-8

**Published:** 2020-11-20

**Authors:** Renyu Ye, Xinsheng Liu

**Affiliations:** 1 State Key Laboratory of Mechanics and Control of Mechanical Structures, Institute of Nano Science and Department of Mathematics, Nanjing University of Aeronautics and Astronautics, Nanjing, 210016 China; 2 School of Mathematics and Physics, Anqing Normal University, Anqing, 246133 China

**Keywords:** Neuroscience, Decision, Perception, Neural decoding

## Abstract

This paper investigates the influence of a known cue on the oblique effect in orientation identification and explains how subjects integrate cue information to identify target orientations. We design the psychophysical task in which subjects estimate target orientations in the presence of a known oriented reference line. For comparison the control experiments without the reference are conducted. Under Bayesian inference framework, a cue integration model is proposed to explain the perceptual improvement in the presence of the reference. The maximum likelihood estimates of the parameters of our model are obtained. In the presence of the reference, the variability and biases of identification are significantly reduced and the oblique effect of orientation identification is obviously weakened. Moreover, the identification of orientation in the vicinity of the reference line is consistently biased away from the reference line (i.e., reference repulsion). Comparing the predictions of the model with the experimental results, the Bayesian Least Squares estimator under the Variable-Precision encoding (BLS_VP) provides a better description of the experimental outcomes and captures the trade-off relationship of bias and precision of identification. Our results provide a useful step toward a better understanding of human visual perception in context of the known cues.

## Introduction

The identification of orientation is a basic visual cognitive task for humans and some animals. The cardinal (i.e., vertical or horizontal) orientations are perceived with higher accuracy than the oblique ones. This visual neuropsychological phenomenon is called the oblique effect^1,2^. That is to say, the cardinal orientations can be estimated by the observers with smaller squared or absolute error than the oblique orientations. The oblique effect is the best-known examples of vision perception and it has been confirmed to exist in a great variety of tasks in visual cognition^3,4^. The oblique effect has underlying neural mechanisms. There are more neurons responding to cardinal orientations than to the oblique orientations in the primary visual cortex^5,6^. Significant anisotropy in human visual cortical activity is discovered through visual cortical fMRI^7^. There are two types of oblique effects: the merely related to low-level visual processing in the primary visual cortex (type I) and the higher-level oblique effect enhancing cognitive processes (e.g. remembering, categorizing or identifying) (type II)^8,9^. The oblique effect occurs after binocular fusion and depends on perceived, rather than physical orientation^10^. The perception results belong to the "type II" oblique effect in a fine-identification task^11^.

Many studies on orientation perception show that identification outcomes have some characteristics. Westheimer found that there is a prominent oblique effect in the two-interval forced choice orientation judgments. And the oblique effect also appears in some visual tasks involving widely-separated elements^12^. In reproducting angled lines experiment, these errors of perceived orientations show a sinusoidal variation and reproducted orientations always tend toward the nearest 45° oblique^13^. The magnitude of the deviation from the cardinal orientations has a non-monotonic pattern and exhibit repulsive biases away from the cardinal orientations in an orientation-estimation task^14^. In an orientation-estimation task, the subjects’ discriminability is best at the cardinals, showing that orientation judgements are more accurate at cardinal (horizontal and vertical) orientations^15^. And the perceptual bias toward oblique orientations was reduced when oriented stimuli were more uncertain^15,16^.

Moreover, the hidden reference axes or explicit references often affect perceptual outcomes in visual identification^11,12,16–21^. Specially, based on local contours, the observers may form an implicit axis (i.e., reference axes) in a visual task. It is assumed that the subjects use the cardinal directions as implicit “reference axes”^17,18^. In general, gravitational, egocentric and visual reference frames may be considered in the visual task^3^ and the three frames are often aligned^19^. In a fine direction discrimination task using moving random-dot stimuli, the subjects reported whether the direction of motion was clockwise or counterclockwise of a decision boundary indicated by a bar outside the edge of the dot-field. The observers’ perception of the direction of motion is biased away from the boundary (the unknown explicit reference)^20^. In forced-choice orientation discrimination, when the reference and the stimulus are presented simultaneously and there is a sufficient distance between them to avoid orientation interaction, the oblique effect of discrimination is reduced considerably^12^. Rauber and Treue conducted an estimation task of direction, in which an unknown-direction line segments presented simultaneously with the moving random-dot pattern at the edge of motion area. There are the robust systematic biases which deviate away from the nearest horizontal cardinal direction. Their results also show that the subjects may have selected different references and their values to estimate goals according to experimental tasks and stimuli^18^. Wiese and Wenderoth^21^ have reproduced Rauber and Treue’s experiments in which the moving dots and the reference line presented simultaneously. Their experiment results also exhibit reference repulsion from the horizontal cardinal directions. But there is no unified statement about the influnce of the references. Zamboni et al. asked subjects estimated the direction of motion in the presence or absence of unknown reference lines. The unknown reference line was randomly chosen for each trial and presented at the edge of a circular aperture. The estimated mean directions were influenced by the position of the reference line^11^. Patten et al. used experiments to explore the behavioral and neural consequences of manipulating stimulus certainty in the context of orientation processing. The reference line on outside of the stimulus was chosen to prevent a biased representation of cardinal and primary oblique orientations and was not used as an independent variable^16^.

Meanwhile, the congnitive process of cues integration is a higher-level process and the vision perception can be described as a Bayesian inference process^11,15,22–24^. Bayesian inference theories successfully explicate many visual neuropsychological phenomena^25–29^. The Bayesian inference framework provides the probabilistic way in which the target orientations are optimally estimated by integrating current sensory input and prior knowledge of these targets^30^. Once there exist the contextual cues in the visual task, humans can integrate available information of cues to achieve the optimal or suboptimal perception results in a complex environment, such as in sensorimotor learning^26^. Based on the framework of Bayesian inference, Ma and Jazayeri^27^ studied how subjects integrate the contextual cues information to obtain measurements of task. Furthermore, Ma et al.^31^ and Rich et al.^32^ concluded that Bayesian inference can be implemented through the probabilistic population coding model.

Although there are many studies on orientation identification, the psychophysical experiments and the involving Bayesian orientation identification model under the condition that the known reference line intersects with the target has not been considered in previous studies. Here we investigate the influence of a known oriented reference line on orientation identification, and provide an explanation of the identification outcomes under the Bayesian inference framework. To address this issue, we design the orientation identification task. Eleven participants completed the orientation identification task with and without reference line. The experimental results suggest that the known tilted reference line significantly affects the identification accuracy and the oblique effect of orientation identification is obviously weakened.

We then provide an explanation of the identification outcomes in the presence of the reference line under the Bayesian inference framework. We model the orientation identification as a probabilistic encoding–decoding process. The Equal-Precision encoding (the same precision across stimuli and trials) and Variable-Precision encoding (precision vary across stimuli) patterns are considered in the encoding process. Comparing the predictions of model to the experimental data, we find that the data are best explained by Bayesian Least Squares estimator under the Variable-Precision (BLS_VP) encoding pattern.

## Materials and methods

### Ethics statement

The procedures were carried out in accordance with the National Commission for the Protection of Subjects of Behavioral Research guidelines for human studies. Before the study was initiated, informed consent was acquired for each subject and approval was obtained from the local Ethics Committee of Nanjing University of Aeronautics and Astronautics (IRB number 201807).

### Participants

Eleven subjects participated in the orientation identification task (four females and seven males; age range 18–28 years old). All of them have normal or corrected-to-normal visions and they were naive to the purpose of the experiments.

### Apparatus and stimuli

Line orientation stimuli and a known reference line were presented on a 21 in. liquid crystal display (LCD) with 120 Hz refresh rate and 1024 × 768 pixel resolutions. In order to suppress the influence of the edges of the screen on identification, all stimuli were presented in a central gray disk with 400 pixels in radius. Luminance was set to the middle range (16 cd/m^2^). We kept the room’s lights off for the entire duration of each trial session. Subjects’ head positions were controlled using a chin and forehead rest located 54 cm away from the computer screen. The height was individually adjusted to hold the subjects’ eyes level to the center of the screen in order to ensure the gravitational, egocentric or body-centered are aligned. The PsychoPy software package^33^ was used to generate the stimuli and the reference line, and record timing and the estimates. The target stimulus was a green line (length 500 pixels; width 2 pixels; RGB [0, 255, 0]) and the reference line was a light gray dashed line (length 200 pixels; width 1 pixel; RGB [200, 200, 200]).

We chose the orientation of reference line at 45°. This is because the identification precisions of five oblique orientations (i.e., 15°, 30°, 45°, 60° and 75°) were lower than the precision of cardinal orientations^34^. And the oblique orientations near the 45° reference line may be least affected by the cardinal direction. We did the similar preliminary experiments of orientation identification. The results of preliminary experiments showed there is no significant difference in the identification precision in the vicinity of the cardinal orientations under two situations (reference presence and absence) (Supplementary Tables S1, S2 and Fig. S1). So we reduced the range of orietations only by looking at the orientation near the reference line. The stimulus orietations were selected randomly from Uniform (22, 68). And sampling the stimuli from the interval [22°, 68°] can decrease the impact of hidden cardinal contour on identification, since the edges corresponding to these stimulus angles are relatively far away from the horizontal and vertical axes. The subjects can use the cardinal (horizontal/vertical) directions as implicit “reference axes”^19,35^. For convenience, we chose 25 target stimulus orientations (22°, 24°, …, 44°, 45°, 46°, 48°, …, 68°) from the interval [22°, 68°]. Each of the 25 values was repeated 16 times, for a total of 400 trials.

### Experimental procedure

**Figure 1:**
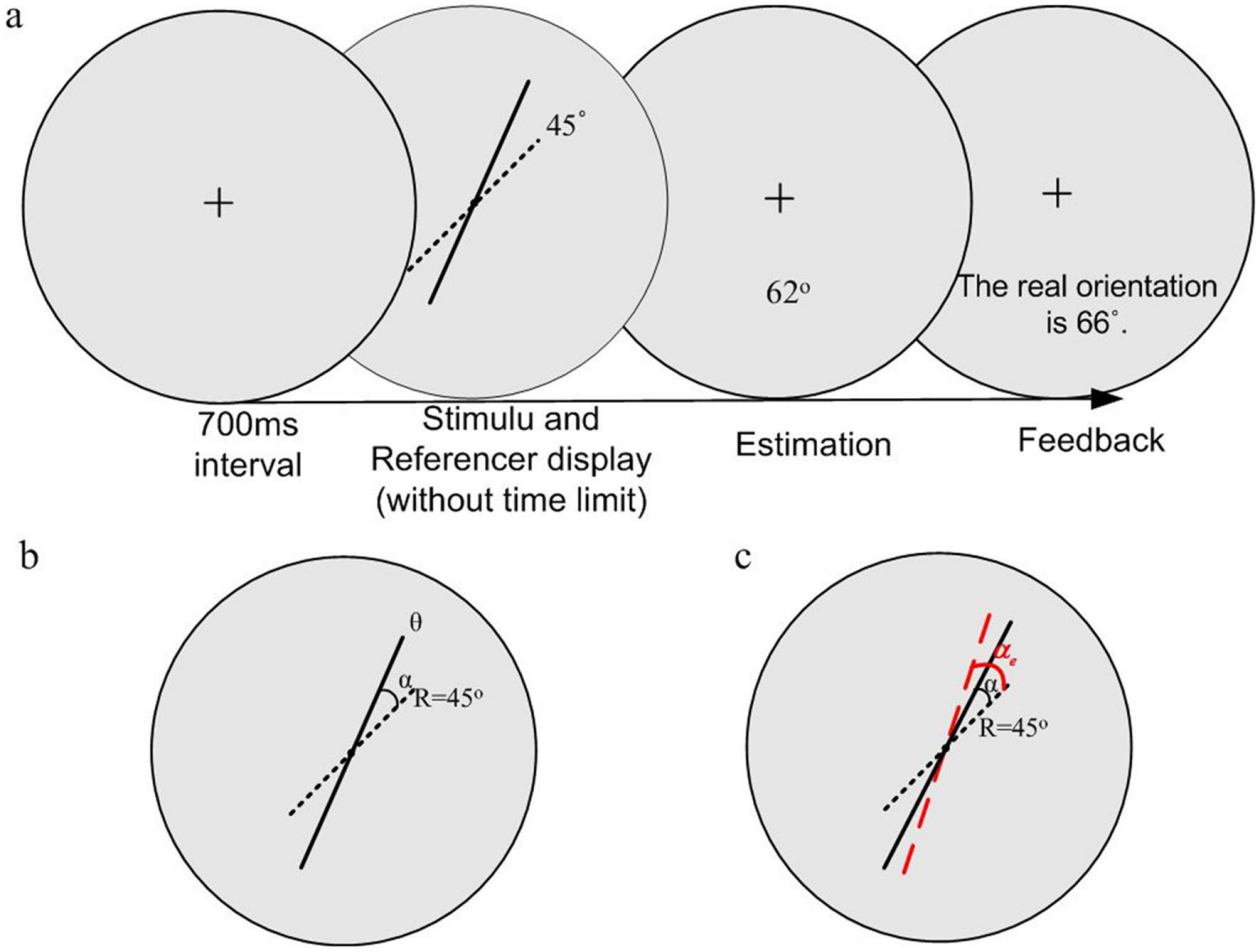
The orientation identification task in the presence of the 45° reference with the correct feedback. (**a**) Procedure of orientation identification task with the correct feedback in the learning phase. The trial started with the appearance of a central point presented in the gray disk. After 700 ms interval, a target stimulus (solid line) was displayed along with a 45° tilted reference line (dotted line) in the middle of the screen without time limit. Subjects entered the numbers of estimate for the target orientation via the keyboard. Then the true orientation values were given, followed by 700 ms interval. (**b**) The cognitive angle. The angle between the target line and the known tilted reference line is called the cognitive angle and is denoted as $\alpha$. The orientation of the stimulus and the known reference are denoted as $\theta$ and R respectively. (**c**) Reference repulsion: the small cognitive angles $\alpha$ have a tendency to overestimate so that there are systematic biases away from the reference line.

In this orientation identification task, the subjects identified a target orientation and entered estimated value via the keyboard. Each trial started with the appearance of a central fixed point in the gray disk for 700 ms. Then the target stimulus line and the known reference line were displayed simultaneously on the screen (Fig. 1a). The subjects were instructed to estimate and input the values of target orientations via the keyboard (if making a mistake, the subjects can press R to re-enter). The estimated values were recorded.

To ensure that the subjects have enough time to adapt their responses to the range of target orientations and further obtain the prior knowledge of target orientations, each subject performed 400 trials before the experiment, which is called the Learning phase. In the Learning phase, the correct feedback of each stimulus was provided after the estimate was input, followed by 700 ms time interval (Fig. 1a). After completing all trials in the learning phase, each subject carried out the orientation identification experiments without feedback, which is called the Estimating phase. Each subject completed 800 trials in the estimating phase. These two phases of psychophysical tests were performed on different days. The subjects first performed control experiment without the known reference line. Other than the presence of the known reference line, the procedure of control experiment is the same as the one with reference. Prior to the experimental trials, each subject was informed about the degree of the reference line and underwent some practice to familiarize oneself with the tasks and procedure. The subjects were asked to rest for at least 10 s when the experiments were carried out in half. There was no time limit to identify a target stimulus for each subject.

### Data analysis

To evaluate the perceptual performance in each situation, we first calculated three metrics for each target orientation separately. These three metrics are defined as follows.

1. Identification bias (BIAS): the mean of the estimation errors of orientations. That is, the bias of the *j* th target stimulus $s_{j}$ is $BIAS_{j} = \sum\nolimits_{{{\text{i}} = 1}}^{n} {{{\left( {x_{ji} - s_{j} } \right)} \mathord{\left/ {\vphantom {{\left( {x_{ji} - s_{j} } \right)} n}} \right. \kern-\nulldelimiterspace} n}}$, where $x_{ji}$ is the *i* th estimate of $s_{j}$ and *n* is the number of the stimulus $s_{j}$ appears in the experiment. The value BIAS is signed and reflects the degree to which a subject’s estimates deviates on average from the true stimulus value. If the sign is positive, it means that the true stimulus is overestimated, and vice versa.
2. Standard deviation of identification (SD): The SD value of $s_{j}$ is $SD_{j} = \sqrt {\sum\nolimits_{{{\text{i}} = 1}}^{n} {{{\left( {x_{ji} - \overline{x}_{j} } \right)^{2} } \mathord{\left/ {\vphantom {{\left( {x_{ji} - \overline{x}_{j} } \right)^{2} } n}} \right. \kern-\nulldelimiterspace} n}} }$, where $\overline{x}_{j} = \sum\nolimits_{i = 1}^{n} {x_{ji} /n}$ is the mean of estimates for $s_{j}$. The SD value reflects the variability of the estimated orientations. The smaller the value SD, the higher the identification precision of the estimates, and vice versa.
3. Mean squared error of identification (MSE): The MSE of $s_{j}$ is $MSE_{j} = \sum\nolimits_{{{\text{i}} = 1}}^{n} {{{\left( {x_{ji} - s_{j} } \right)^{2} } \mathord{\left/ {\vphantom {{\left( {x_{ji} - s_{j} } \right)^{2} } n}} \right. \kern-\nulldelimiterspace} n}}$. The value MSE is a measure of the overall quality of orientation identification. The smaller the value MSE, the better the quality, and vice versa.

In each situation with or without reference line, we computed these three metrics for each of 25 stimulus orientations (referred to by subscript j): $BIAS_{j} ,SD_{j} \;and\;MSE_{j} \;(j = 1, \ldots ,25).$ The three metrics satisfied: $MSE_{j} = BIAS_{j}^{2} + SD_{j}^{2} \;(j = 1, \ldots ,25).$ The overall BIAS and SD of single subject’s data are defined as: $BIAS = \sqrt {\sum\nolimits_{j = 1}^{25} {{{BIAS_{j}^{2} } \mathord{\left/ {\vphantom {{BIAS_{j}^{2} } {25}}} \right. \kern-\nulldelimiterspace} {25}}} }$ and $SD = \sqrt {\sum\nolimits_{{{\text{j}} = 1}}^{25} {{{SD_{j}^{2} } \mathord{\left/ {\vphantom {{SD_{j}^{2} } {25}}} \right. \kern-\nulldelimiterspace} {25}}} } .$ The corresponding overall mean squared error of identification is defined as: $MSE_{S} = BIAS^{2} + SD^{2}$, which highlights the trade-off between bias and identification precision. We computed these three metrics for all subjects’ data. A repeated-measures two-way ANOVA was used to determine any significant influences of the reference on the identification outcomes.

### Bayesian cue integration model of orientation identification

**Figure 2:**
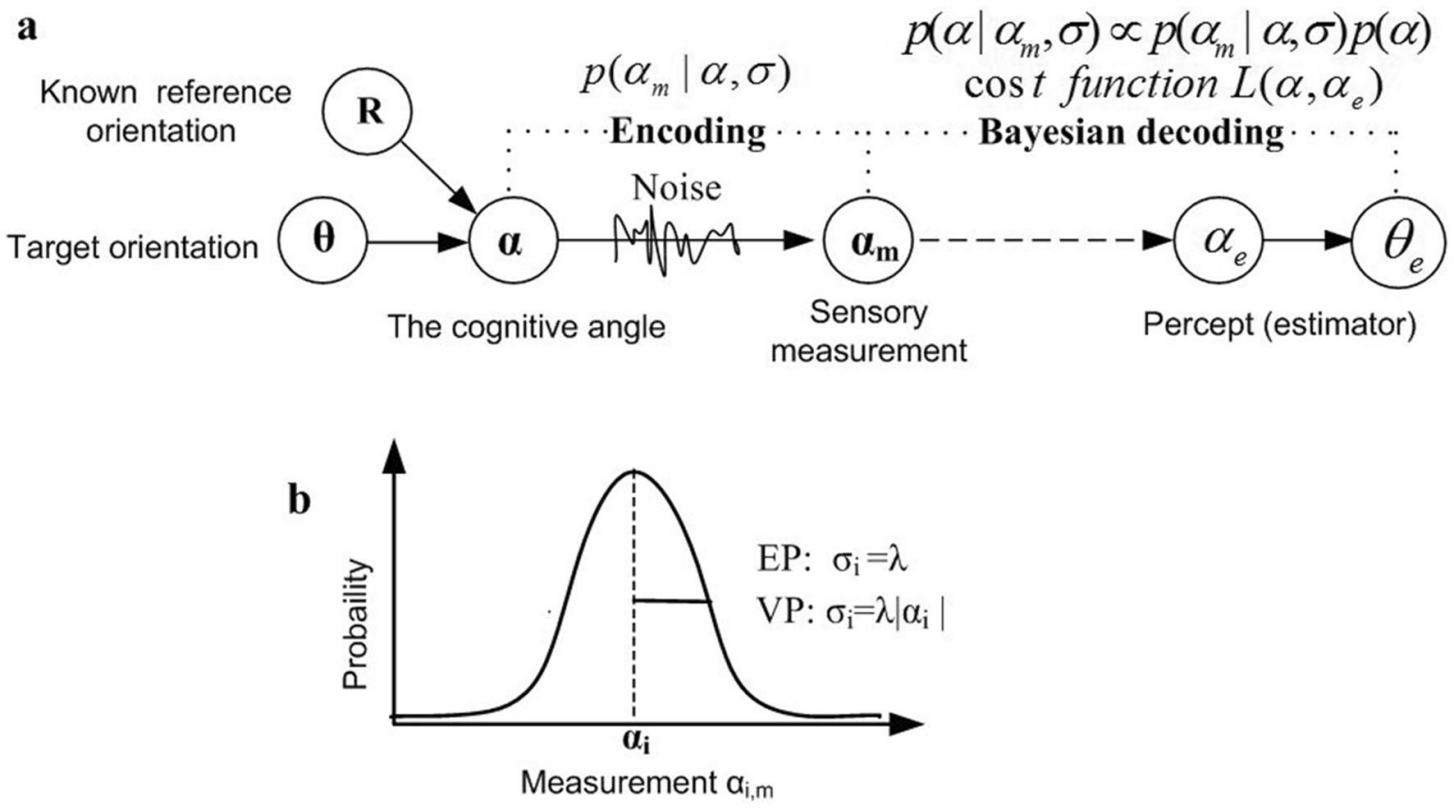
Bayesian cue integration model of orientation identification in the presence of a known reference. (**a**) Diagram of the Bayesian inference process of orientation identification in the presence of a known reference. The process consists of the encoding stage and the Bayesian decoding stage. In the encoding stage, because of sensory measurement noise, the cognitive angle $\alpha$ is encoded as a internal visual sensory measurement or evidence,$\alpha_{m}$, which is characterized by the conditional probability distribution, $p(\alpha_{m} |\alpha ,\sigma )$, where $\sigma$ is the standard deviation of inter visual sensory measurement. In the Bayesian decoding stage, the angle estimator $\alpha_{e}$ is specified based on the posterior distribution $p(\alpha |\alpha_{m} ,\sigma )$ and the cost function $L(\alpha ,\alpha_{e} )$. The estimator of target orientation $\theta_{e}$ is equal to the sum of $\alpha_{e}$ and the known reference orientation *R*. (**b**) Two types of encoding precision of internal visual sensory measurement. Equal-Precision encoding (EP): $p(\alpha_{m} |\alpha_{i} ,\sigma ) = N(\alpha_{m} ;\alpha_{i} ,\lambda^{2} ).$ The conditional distribution has a fixed standard deviation for all cognitive angles. Variable-Precision encoding (VP): $p(\alpha_{m} |\alpha_{i} ,\sigma ) = N(\alpha_{m} ;\alpha_{i} ,(\lambda \left| {\alpha_{i} } \right|)^{2} ).$ The standard deviation grows linearly with its mean,$\sigma_{i} = \lambda |\alpha_{i} |$ ($\lambda$ refer to as the Weber fraction).

To quantitatively explain the psychophysical results, we put forward a cue integration model based on the Bayesian inference framework. The subjects are assumed to be Bayesian observers who can optimally estimate target orientations in a noisy environment by integrating prior knowledge of target orientations and current sensory input^26^. The identification process of orientation is modeled as Bayesian probabilistic inference, and consists of an encoding stage and a Bayesian decoding stage^36,37^ (Fig. 2a). We assume that the observers consider the known reference line as a distinct sensory cue, which provides the subjects with lots of additional contextual information. Let $\theta$ be the target orientation, and $R = 45^{{\text{o}}}$ be the known reference’s orientation. Define $\alpha = \theta - R$ as the cognitive angle **(**Fig. 1b). Thus, the estimation of a target orientation is transformed into the estimation of an cognitive angle $\alpha$.

In the encoding stage, as a result of sensory noise, the subjects obtain the cognitive angle’s sensory measurement or evidence $\alpha_{m}$, which may differ from the true angle $\alpha$. The primary visual cortex implements the encoding process in brain^38^. Repeated appearance of the same target orientation resulted in slightly different measurements, which could be described by a conditional probability distribution $p(\alpha_{m} |\alpha ,\sigma )$. We model the conditional probability distribution of sensory measurement as a Gaussian distribution with mean of $\alpha$ (Fig. 2b). Two types of encoding precision of the conditional probability distribution are considered^39–41^:

- Equal-Precision (EP) encoding (Fig. 2b), in which sensory measurements have the same precision across trials and stimuli, $\sigma = \lambda$. In this case, the conditional probability distribution of sensory measurement $\alpha_{m}$ can be denoted by $p_{EP} (\alpha_{m} |\alpha ,\lambda ) = N(\alpha_{m} ;\;\alpha ,\lambda^{2} )$.
- Variable-Precision (VP) encoding (Fig. 2b), in which sensory measurement precision vary across stimuli. We take the standard deviation grows linearly with its mean, $\sigma = \lambda |\alpha |$ (see Supplementary Fig. S2), where $\lambda$ is referred as the Weber fraction associated with the measurement. The characteristic of the encoding is called scalar variability^42–44^. Then, $p_{VP} (\alpha_{m} |\alpha ,\lambda ) = N(\alpha_{m} ;\;\alpha ,\lambda^{2} \alpha^{2} )$.

In the decoding stage, the Bayesian observer can combine with the prior probability distribution of target angle $p(\alpha )$ and the conditional probability of sensory measurement $p(\alpha_{m} |\alpha ,\lambda )$ to compute the posterior distribution of target angle $p(\alpha |\alpha_{m} ,\lambda )$ (Fig. 2a). Then, given a cost function $L(\alpha ,\alpha_{e} )$ which quantifies the cost of estimating true orientation $\alpha$ as $\alpha_{e}$, the observer obtains the Bayesian estimator $\alpha_{e}$ of true angle $\alpha$ by minimizing the expected posterior risk. According to the relationship between the target orientation $\theta$ and the cognitive angle $\alpha$, the estimator of the stimulus orientation is derived, that is, $\theta_{e} = R + \alpha_{e}$.

To estimate the cognitive angle, the subjects need to compute the posterior distribution of target orientation $p(\alpha |\alpha_{m} ,\lambda )$ using Bayes’ rule as follows:

1 $$p(\alpha |\alpha_{m} ,\lambda ) = \frac{{p(\alpha_{m} |\alpha ,\lambda )p(\alpha )}}{{\int {p(\alpha_{m} |\alpha ,\lambda )p(\alpha )d\alpha } }} = \left\{ {\begin{array}{*{20}c} {\frac{{p(\alpha_{m} |\alpha ,\lambda )}}{{\int_{{\alpha_{\min } }}^{{\alpha_{\max } }} {p(\alpha_{m} |\alpha ,\lambda )d\alpha } }},} & {\alpha_{\min } \le \alpha \le \alpha_{\max } } \\ {0,} & {otherwise} \\ \end{array} } \right.$$

where $\alpha_{\min }$ and $\alpha_{\max }$ are the minimum and maximum value of supporting set of prior distribution of cognitive angle, respectively. The prior distribution of cognitive angle $p(\alpha )$ is a uniform distribution.

Given an objective cost function $L(\alpha ,\alpha_{e} )$, Bayesian estimator of the angle $\alpha$ is chosen to minimize the expected cost averaged over the posterior distribution of target orientation. According to the relationship of the cognitive angle and target orientation, the observer could infer the estimator of target orientation,

2 $$\theta_{e} = R + \alpha_{e} = R + \mathop {argmin}\limits_{{\alpha_{e} }} \int {L(\alpha ,\alpha_{e} )p(\alpha |\alpha_{m} ,\lambda )d\alpha \overset{\wedge}{=}R + g(\alpha_{m} ,\lambda )} .$$

When the subjects chose the cost function $L(\alpha ,\alpha_{e} ) = - \delta (\alpha - \alpha_{e} )$, where $\delta ( \cdot )$ denotes the Dirac delta function, the corresponding Bayesian estimator is obtained by maximizing a posteriori (MAP), then

3 $$\theta_{e,MAP} \hat{ = }R + \alpha_{e,MAP} = R + \mathop {argmax}\limits_{\alpha } p(\alpha |\alpha_{m} ,\lambda ).$$

Under Equal-Precision (EP) encoding, the conditional probability of sensory measurement is $p_{EP} (\alpha_{m} |\alpha ,\lambda ) = N{\kern 1pt} {\kern 1pt} (\alpha_{m} ;\alpha ,\lambda^{2} )$. Then, the Maximum posteriori estimator is denoted by

4 $$\theta_{e,MAP\_EP} \hat{ = }R + \alpha_{e,MAP\_EP} = \left\{ {\begin{array}{*{20}l} {R + \alpha_{\min } ,} \hfill & {\alpha < \alpha_{\min } } \hfill \\ {R + \alpha_{m} ,} \hfill & {\alpha_{\min } \le \alpha \le \alpha_{\max } } \hfill \\ {R + \alpha_{\max } ,} \hfill & {\alpha > \alpha_{\max } } \hfill \\ \end{array} } \right..$$

Under Variable-Precision (VP) encoding, the conditional probability of sensory measurement is $p_{VP} (\alpha_{m} |\alpha ,\lambda ) = N(\alpha_{m} ;\alpha ,\lambda^{2} \alpha^{2} )$ and the Maximum posteriori estimator is a function of $\alpha_{m}$ and $\lambda$, that is,

5 $$\theta_{e,MAP\_VP} \hat{ = }R + \alpha_{e,MAP\_VP} = \left\{ {\begin{array}{*{20}l} {R + \alpha_{\min } ,} \hfill & {\alpha < \alpha_{\min } } \hfill \\ {R + \alpha_{m} \left[ {\frac{{ - 1 + \sqrt {1 + 4\lambda^{2} } }}{{2\lambda^{2} }}} \right],} \hfill & {\alpha_{\min } \le \alpha \le \alpha_{\max } } \hfill \\ {R + \alpha_{\max } ,} \hfill & {\alpha > \alpha_{\max } } \hfill \\ \end{array} } \right..$$

When the cost function is the square error between the estimate and the stimulus, $L(\alpha ,\alpha_{e} ) = (\alpha - \alpha_{e} )^{2}$, the corresponding Bayesian estimator is the mean of the posterior distribution of the angle which is called Bayesian least squares (BLS) (See Appendix A),

6 $$\theta_{e,BLS} \hat{ = }R + \alpha_{e,BLS} = R + \int {\alpha p(\alpha |\alpha_{m} ,\lambda )\;d\alpha } = R + \frac{{\int_{{\alpha_{\min } }}^{{\alpha_{\max } }} {\alpha p(\alpha_{m} |\alpha ,\lambda )\;d\alpha } }}{{\int_{{\alpha_{\min } }}^{{\alpha_{\max } }} {p(\alpha_{m} |\alpha ,\lambda )\;d\alpha } }}.$$

Under the EP and VP encoding, we can also obtain the corresponding Bayesian estimators $\theta_{e,BLS\_EP}$ and $\theta_{e,BLS\_VP}$, respectively. The two estimators have no analytical expressions because integrals of Eq. (6) are not analytically solvable. We can obtain the approximate values of two estimators using the Simpson rule to compute the two integrals in Eq. (6).

### Parameter estimation of model

Based on the experimental data in the presence of reference line, the maximum-likelihood estimates of parameter of model are obtained using Monte Carlo simulations. There is one parameter $\lambda$ in model: the standard deviation of the sensory measurement distribution, $\sigma = \lambda$ (equal precision pattern), or Weber fraction, $\sigma = \lambda |\alpha |$ (variable precision pattern). We wish to find the most probable parameter value which maximizes the likelihood function given the psychophysics experimental data set. Let $\alpha^{j}$ be the j-th cognitive angle stimuli and $\alpha_{e}^{ji}$ be the i-th estimate of $\alpha^{j}$. The experimental data set is expressed as $\left\{ {(\alpha^{j} ,\alpha_{e}^{ji} )} \right\}_{i = 1, \ldots ,n}^{j = 1, \ldots ,k}$, where $k$ is the number of target orientations and $n$ is the number of $\alpha^{j}$ appears in the experiment. The joint conditional probability of $\left\{ {\alpha_{e}^{ji} } \right\}_{i = 1, \ldots ,n}^{j = 1, \ldots ,k}$ is served as a likelihood function of parameter $\lambda .$ Assuming that the experimental data $\left\{ {\alpha_{e}^{ji} } \right\}_{i = 1, \ldots ,n}^{j = 1, \ldots ,k}$ associated with any angle $\alpha$ are conditionally independent, the likelihood function can be expressed as the product of their individual conditional probabilities as follows:

7 $$L_{M} (\lambda ;data) = \prod\limits_{j = 1}^{k} {p\left( {\alpha_{e}^{j1} ,\alpha_{e}^{j2} , \ldots ,\alpha_{e}^{jn} |\alpha^{j} ,M,\lambda } \right)} = \prod\limits_{j = 1}^{k} {\prod\limits_{i = 1}^{n} {p(\alpha_{e}^{ji} |\alpha^{j} ,M,\lambda )} } ,$$

where *M* denotes the one of four types of the estimators. By taking the logarithm of both sides, we can obtain

8 $$\log L_{M} (\lambda ;data) = \sum\limits_{j = 1}^{k} {\sum\limits_{i = 1}^{n} {\log p(\alpha_{e}^{ji} |\alpha^{j} ,M,\lambda )} }$$

Based on the conditional independence of Bayesian network, the variables $(\alpha ,\lambda )$ and the estimator of angle $\alpha_{e}$ are conditionally independent given sensory measurement $\alpha_{m}$. The conditional probability $p(\alpha_{e} |\alpha ,\lambda )$ can be expressed as

9 $$\begin{aligned} p(\alpha_{e} |\alpha ,M,\lambda ) & = \int {p(\alpha_{e} |\alpha_{m} ,M)p(\alpha_{m} |\alpha ,\lambda )\;d\alpha_{m} } \\ & = \int {\delta (\alpha_{e} - g_{M} (\alpha_{m} ,\lambda ))p(\alpha_{m} |\alpha ,\lambda )\;d\alpha_{m} } , \\ \end{aligned}$$

where $\delta ( \cdot )$ denotes the Dirac delta function.

Since the probability in Eq. (8) cannot be computed analytically, we estimate numerically the sum of the logarithm likelihood function by Monte Carlo simulations^41^. For a given parameter value $\lambda$, type *M* and the *j* th cognitive angle $\alpha^{j} ,$ we perform the following procedure to compute the probability $p(\alpha_{e}^{ji} |\alpha^{j} ,M,\lambda )$: 800 sensory measurement values are randomly drawn from the conditional probability distribution $p(\alpha_{m} |\alpha^{j} ,\lambda )$ and the sensory measurement data set $\{ \alpha_{m}^{j,i = 1, \cdots ,800} \}$ is obtained. Then each $\alpha_{m}^{ji}$ is substituted separately into Eqs. (4), (5) and (6), and the data sets of the corresponding estimates $\{ \hat{\alpha }_{e}^{j,i = 1, \ldots ,800} \}$ are obtained. The probability $p(\alpha_{e}^{ji} |\alpha^{j} ,M,\lambda )$ is approximately equal to the frequency that $\alpha_{e}^{ji}$ appears in $\{ \hat{\alpha }_{e}^{j,i = 1, \ldots ,800} \}$. Thus, the sum of the logarithm likelihood function of experiment data (Eq. 8) can be calculated approximately.

We can find the maximum likelihood estimates of the parameters that maximized Eq. (8). Given subject’s data and the type *M*, we vary the value for $\sigma$ in steps of 0.01 between 1 and 8, and the value for $w$ in steps of 0.01 between 0.1 and 0.4. For each parameter value, we obtain the sum of logarithm likelihood function of experimental data. The parameter value which maximizes Eq. (8) is an approximated value for the maximum likelihood estimate of the parameter.

## Results

### Experimental results

For each subject, except for trials data in the learning phase, we collected 800 trials data in the estimating phase. By analyzing experimental data, the following three features are revealed from the experimental data.

**Figure 3:**
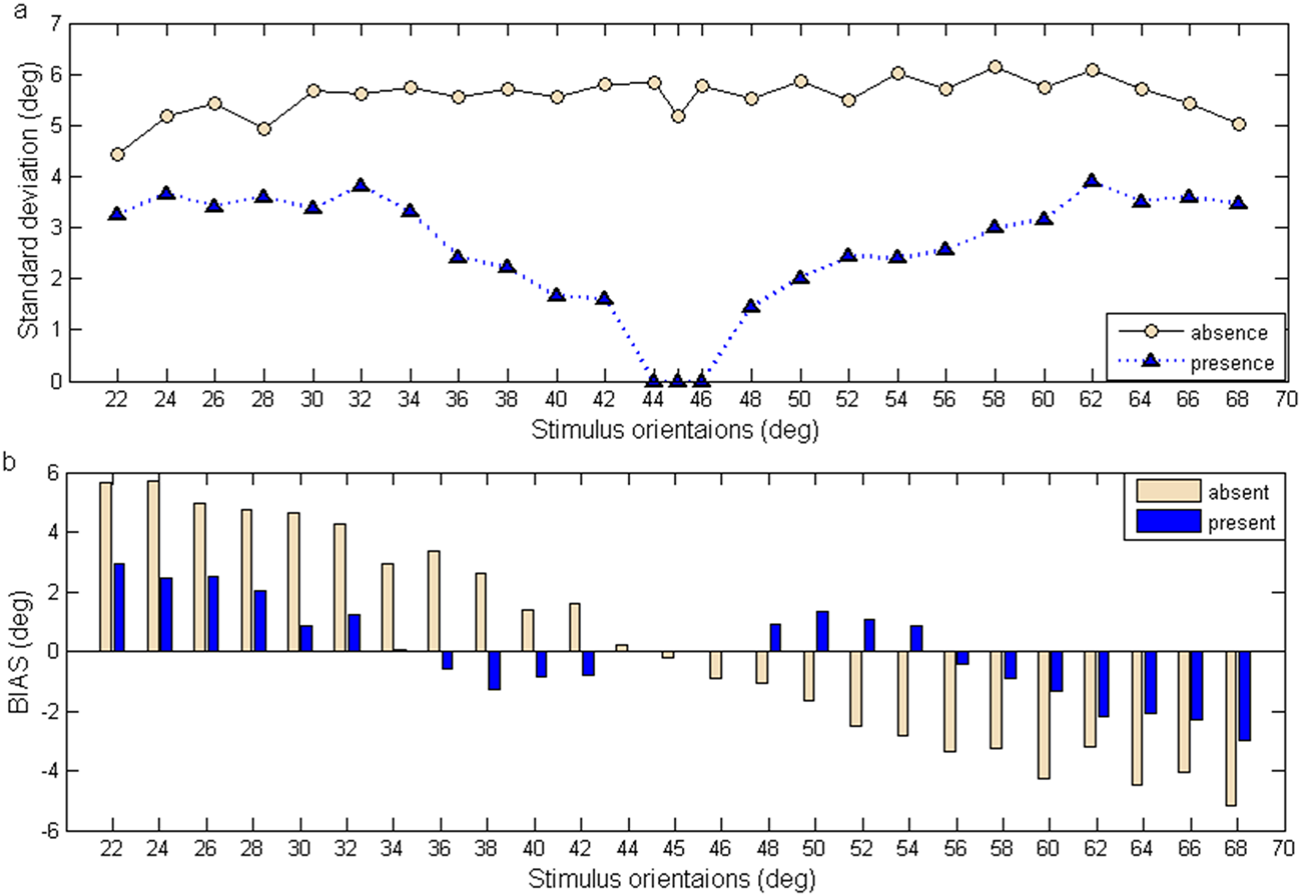
The standard deviation and bias of orientation identification across all subjects in both situations. (**a**) The precision of identification across all subjects as a function of target orientations. The triangles represent the SD values with reference and the dots represent the SD values without reference. (**b**) The contrast histogram for the BIAS of orientation identification across 11 subjects in the two situations.

First, the identification precision is obviously improved when the known reference line is presented (Fig. 3a). In the absence of reference, the SD values of 25 target oblique orientations range from 4.43° to 6.15° and are obviously larger than the cardinal orientations’ SD values (0° (0.501), 90° (1.114)) which is from the results of our preliminary experiment (Supplementary Table S1). In the presence of reference, as the target orientation moves closer to the reference line, the identification precision becomes higher. All subjects can accurately discriminate the stimuli of the 45°, 44° and 46° (Fig. 3a). We make 2 (reference situation) × 25 (orientation) repeated-measures two-way ANOVA for the SD. The results suggest that the known reference line significantly affects the precision of identification ($F_{1,500} = 3{73},\;p < 0.0001$) and the interaction between “reference situation” and “orientations” has significant effects on the SD ($F_{24,500} = 3.57,\;p < 0.0010$).

**Figure 4:**
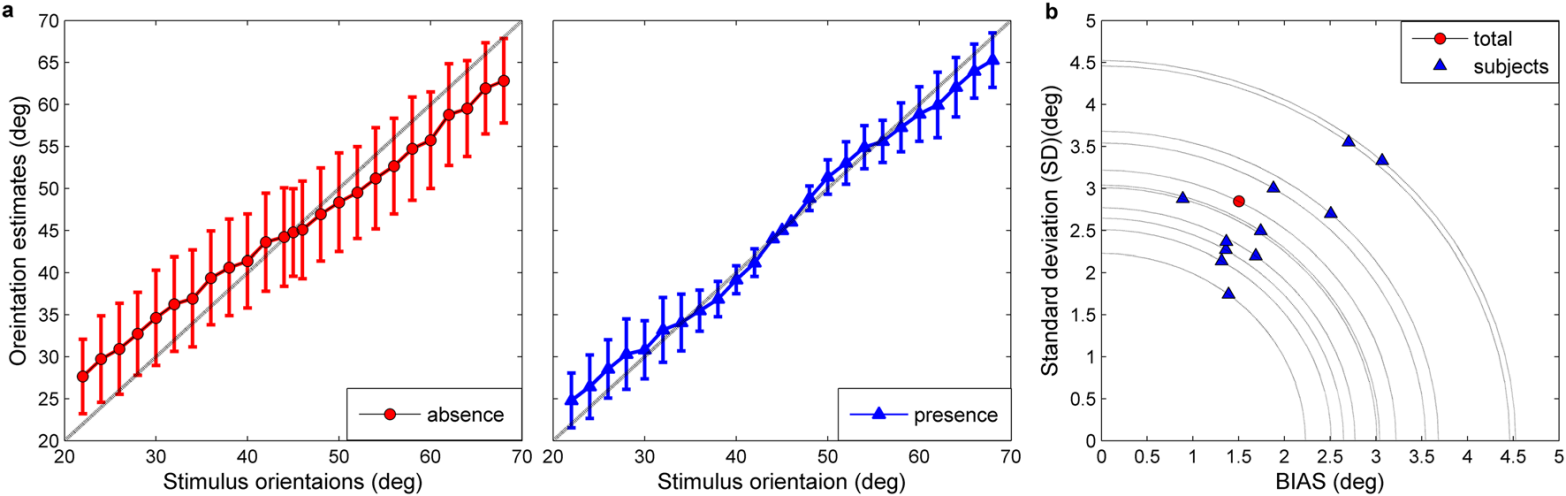
Orientation-identification results across all subjects in the presence of reference. (**a**) The error bar (mean ± 1 standard deviation across eleven subjects) for the 25 stimulus orientations with and without reference. The diagonal (dotted line) indicates that the estimates and target orientations coincide. (**b**) The SD versus BIAS for the data from each individual subject and total data in the presence of reference. Dashed quarter circles show the overall MSE values associated with the corresponding SD and BIAS. There is a trade-off between the BIAS, SD and MSE.

Second, the biases of identification across all subjects in two situations present different characteristics. The known reference line significantly affects the BIAS ($F_{1,500} = 4,\;p = 0.0455$). When the reference line is absent, the stimuli of less than 45° are overestimated and the ones more than 45° are underestimated (Figs. 3b, 4a left panel). The phenomenon is known as the regression effect^45–47^. In the presence of reference, the reference line divides the stimulus interval into two parts and there exist two-regression effects in the two sub-intervals. Moreover, in the vicinity of the reference line, the stimuli of less than 45° are underestimated, while those over 45° are overestimated (Figs. 3b, 4a right panel). The sign of biases is opposite to the ones without the reference line. The biases of orientation perception show the reference repulsion phenomenon^18^. The biases of orientation identification in the vicinity of the orientation of 45° decrease dramatically. The results of repeated-measures two-way ANOVA illustrates the interaction between reference situation and orientations has significant effects on the BIAS ($F_{24,500} = 106.93,\;p < 0.0001$).

Third, in the presence of reference, experimental data show that there is a reasonable trade-off between the precision and the bias of identification (Fig. 4b). Suppose each subject has stable identification ability (MSE_S_), according to the relationship among the three metrics, the BIAS decreases when the SD increases, and vice versa. Figure 4b shows that the precision of identification tends to be improved by adjusting the bias of estimation. They reasonably consider the relationship of BIAS and SD in an identification task.

In summary, the experimental results show that the known reference line has a remarkable impact on the orientation identification. The findings indicate that the subjects can integrate the information of the known reference into orientation identification. Moreover, the known reference line might be one of the important factors that affect the identification.

### Model fitting and comparison

We put forward a Bayesian cue integration model to account for these identification results. Based on different assumptions of encoding precision of sensory stimulus and cost functions in decoding process, we obtain four Bayesian estimators: MAP_EP (Equal-Precision Maximum posteriori estimator), BLS_EP (Equal-Precision Bayesian least squares estimator), MAP_VP (Variable-Precision Maximum posteriori estimator), BLS_VP (Variable-Precision Bayesian least squares estimator). Each estimator has one parameter: the standard deviation of the sensory measurement distribution, $\lambda = \sigma$ (equal precision pattern), or Weber fraction, $\lambda = w$ (variable precision pattern). For each subject’s data and total data across eleven subjects, the maximum likelihood estimate of parameter ($\sigma$ or $w$) is approximately obtained.

We compare each model’s predictions to the experimental data using the trade-off between the BIAS, SD and MSE. At the same time, we also used the cross-entropy (CE) to compare the four models. It is computed as $CE = - \frac{1}{N}\sum\nolimits_{{\text{i = 1}}}^{N} {\sum\nolimits_{k = 1}^{25} {p_{ik} \log \hat{p}_{ik} } }$, where *N* is the number of samples, *k* is the number of classification labels, and $p_{ik}$ and $\hat{p}_{ik}$ are the actual probability and estimated probability respectively. The values for three metrics and cross-entropy can be found in Supplementary informations (Table S4).

We can also theoretically compute the mean and variance of four Bayesian estimators through the Eqs. (4), (5) and (6). The mean and variance of MAP_EP estimator are as follows:

10 $$E\left( {\theta_{e,MAP\_EP} } \right) = \left\{ {\begin{array}{*{20}l} {R + \alpha_{\min } ,} \hfill & {\alpha < \alpha_{\min } } \hfill \\ {R + \alpha ,} \hfill & {\alpha_{\min } \le \alpha \le \alpha_{\max } } \hfill \\ {R + \alpha_{\max } ,} \hfill & {\alpha > \alpha_{\max } } \hfill \\ \end{array} } \right.,\quad VAR\left( {\hat{\theta }_{e,MAP\_EP} |\theta } \right) = \left\{ {\begin{array}{*{20}l} {\sigma^{2} ,} \hfill & {\alpha_{\min } \le \alpha \le \alpha_{\max } } \hfill \\ {0,} \hfill & {otherwise} \hfill \\ \end{array} } \right..$$

The mean and variance of MAP_VP estimator are as follows:

11 $$E\left( {\left. {\hat{\theta }_{e,MAP\_VP} } \right|\theta } \right) = \left\{ {\begin{array}{*{20}l} {R + \alpha_{\min } ,} \hfill & {\alpha > \alpha_{\max } } \hfill \\ {R + \alpha \left[ {\frac{{ - 1 + \sqrt {1 + 4\lambda^{2} } }}{{2\lambda^{2} }}} \right],} \hfill & {\alpha < \alpha_{\min } } \hfill \\ {R + \alpha_{\max } ,} \hfill & {\alpha_{\min } \le \alpha \le \alpha_{\max } } \hfill \\ \end{array} } \right.,$$

12 $$VAR\left( {\hat{\theta }_{e,MAP\_VP} |\theta } \right) = \left\{ {\begin{array}{*{20}l} {\lambda^{2} \alpha^{2} \left[ {\frac{{ - 1 + \sqrt {1 + 4\lambda^{2} } }}{{2\lambda^{2} }}} \right],} \hfill & {\alpha_{\min } \le \alpha \le \alpha_{\max } } \hfill \\ {0,} \hfill & {otherwise} \hfill \\ \end{array} } \right.$$

The means and variances of BLS_EP and BLS_VP estimators can be numerically approximated using Monte Carlo simulations.

**Figure 5:**
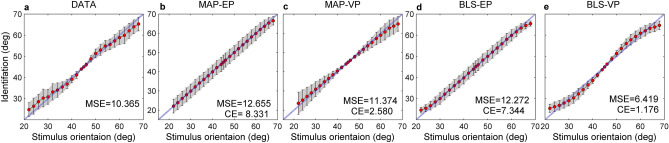
Identification of orientation as a function of stimulus orientation in the presence of reference. (**a**) Circles and error bar: the total data across eleven subjects. Shaded regions are mean ± 1 s.d. The MSE is the mean squared error between subjects’ identification and true stimuli. (**b**–**e**) Circles and error bar for predictions of each Bayesian model. The shaded regions are these predictions (simulated mean ± 1 s.d.). Each MSE is correspondingly the mean squared error between subjects’ identification and the prediction of each model. Each CE is correspondingly the cross entropy.

**Figure 6:**
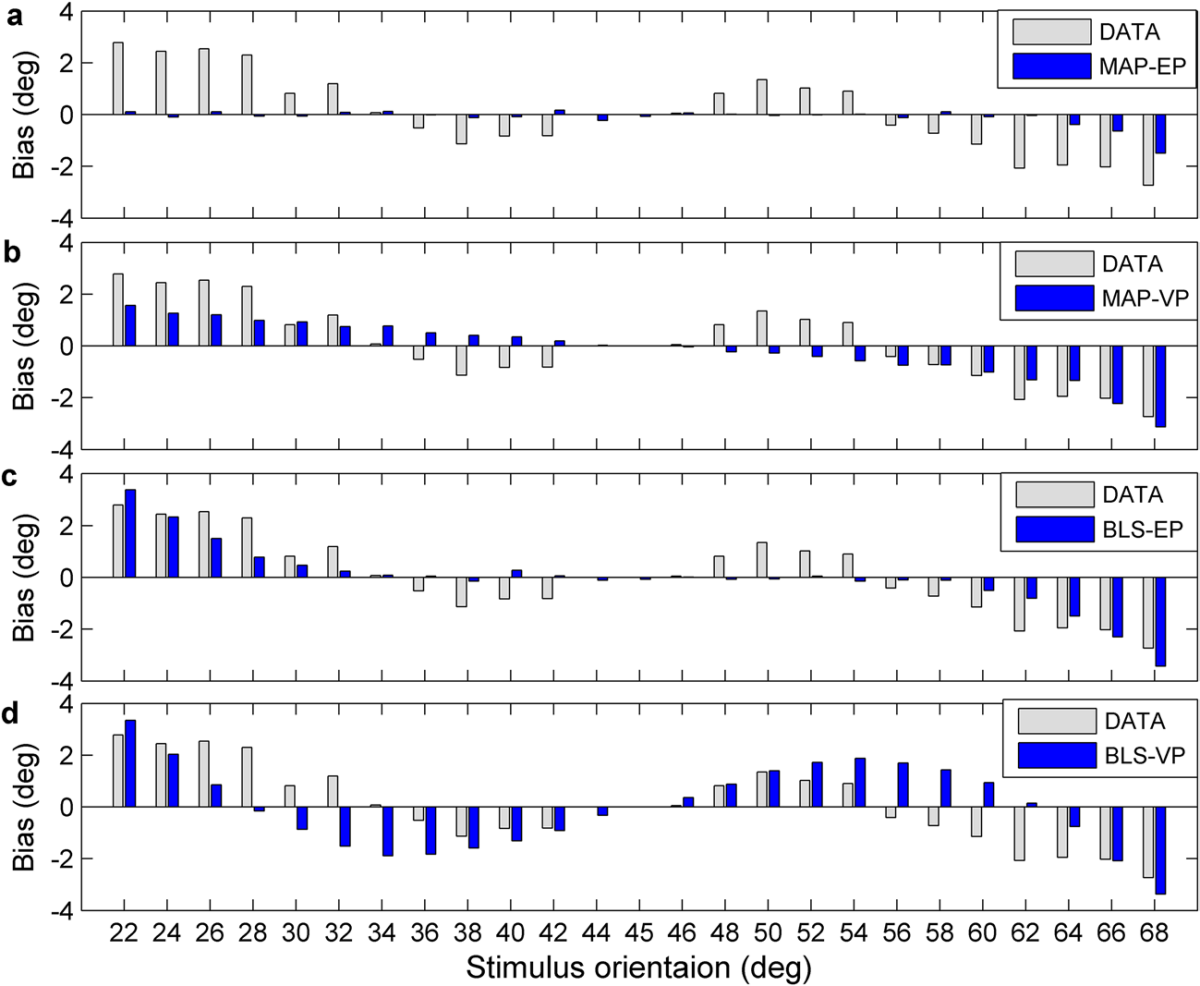
The bias comparison. The contrast histograms for the BIAS of total data and the model predictions are shown.

The Variable-Precision Bayesian models better fit the experimental data than the Equal-Precision models, and the BLS_VP estimator is better than the other three estimators. As we see from the Table 1, the MSE_S_ and CE scores of Variable-Precision estimators obviously are less than that of Equal-Precision counterparts. Figure 5 shows that the Variable-Precision models provide a reasonable description of the precision of experimental data, especially in the vicinity of 45°. Figure 6 displays four contrast histograms for the biases of total data and the biases of model predictions. The MAP_EP estimator is theoretically optimal due to the unbiasedness (Eq. 10), but it clearly fails to capture the characteristic of experimental data (Figs. 5b, 6a). The prediction biases of the MAP_VP and BLS_EP models also fail to account for the phenomenon of reference repulsion (Fig. 6b,c). The BLS_VP model best explains the reference repulsion and two-regression effects of the experimental data (Figs. 5e, 6d).

**Table 1:** The estimates of the parameter and the values for three metrics and cross-entropy baesd on the total data across eleven subjects.

	MLE ($\hat{\lambda }$)	MSE_S_	BIAS	SD	CE
DATA		10.365	1.501	2.848	
MAP_EP	3.6	12.665	0.346	3.541	8.331
MAP_VP	0.27	11.374	1.097	3.189	2.580
BLS_EP	3.75	12.272	1.269	3.265	7.344
BLS_VP	0.23	6.418	1.583	1.978	1.176

MLE is maximum likelihood estimates of parameter for the models. MSE_S_ are the overall mean square errors. The BIAS and SD denote the overall bias and standard deviation of identification errors, respectively (in degrees). CE is the cross-entroy between the actual probability distribution and the predicted probability distribution under each model.

**Figure 7:**
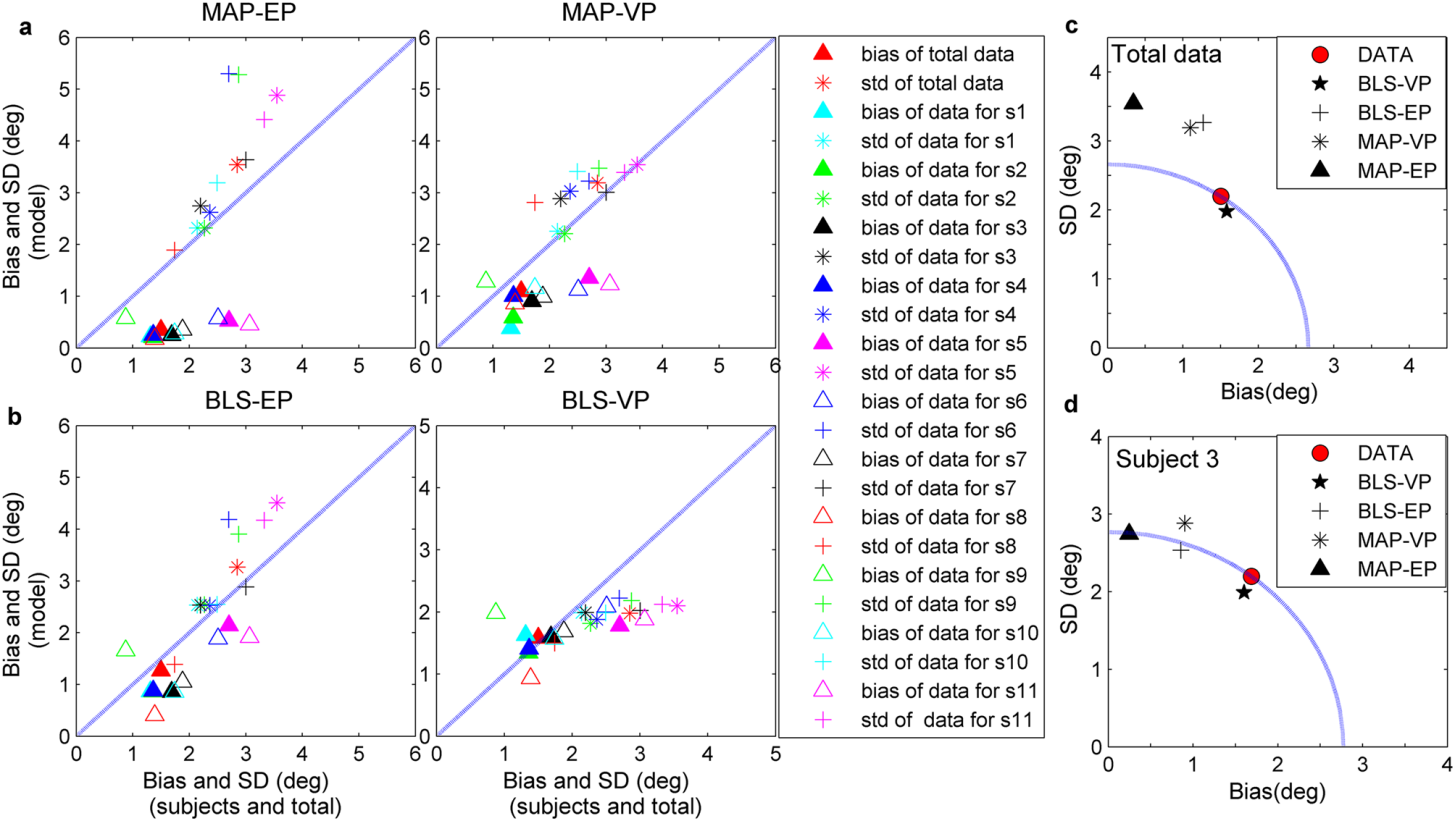
The BIAS and SD of identification for experimental data and model predictions. (**a**) The BIAS (triangle) and SD from the MAP_EP (left) and MAP_VP (right) models as the functions of BIAS and SD computed directly from the data for each subject and total data. Diagonal dashed line represents that the simulated and real data coincide. (**b**) The BIAS and SD from the BLS_EP (left) and BLS_VP models (right). (**c**) The SD versus BIAS for total data in the presence of reference. Dashed quarter circles show that the MSE value associated with the BIAS and SD is 10.365. (**d**) The SD versus BIAS for the data from the third subject in the presence of reference. Dashed quarter circles show that the associated MSE value is 7.682.

Figure 7 illustrates that the BLS_VP model is obviously better than the others at capturing the trade-off relationship between the bias and precision of the data. The BIAS of the MAP estimator are obviously less than that of experimental data and their SDs are larger than that of the data (Fig. 7a,c,d). The BLS_EP model also underestimates the BIAS of orientations and overestimates the SDs of the data across all subjects (Fig. 7b left panel, c,d). However, the BIAS and the SD of the BLS_VP estimator are consistent with the experimental results (Fig. 7b right panel, c,d).

## Discussion

Visual perception can be influenced by contextual cues. In this paper, we have investigated the influence of a known reference line on the orientation identification. We design the experiments in which the subjects estimate the orientations of target lines in the presence of a known reference line, and the control experiments without reference are also conducted. In the absence of reference, the oblique effect and the regression effect exist, which is consistent with the results in the previous studies^34,44,46^.

In the presence of a known reference, the orientation identification performance is obviously improved. The oblique effect is weakened with the improvement of the identification precision of the oblique orientations, especially in the vicinity of the reference line. But the presentation of known reference line may not abolish the type II oblique effect completely. Moreover, we observe the phenomena of two-regression in intervals of (22°, 45°) and (45°, 68°) respectively. A reasonable explanation may be that the stimulus interval is divided into two subintervals by the known reference line, and the orientation identification in the two subintervals respectively presents regression effect. If the orientation of reference is not 45°, the centers of regressions may be correspondingly shifted. Furthermore, there exist robust systematic biases which are deviated from the reference line (i.e., reference repulsion^11,18,20^). The phenomenon may be caused by the overestimate of the angle between the stimulus and the reference.

At the same time, the observer’s visual system can reasonably trade off the relationship of the bias and the precision to improve the overall identification performance. The observer wants to be able to accurately identify target orientations. But in most cases, there exist systematic deviation and uncertainty. We suppose the identification ability is limited (MSE is fixed). The observer may lower the requirements of bias to improve the identification precision. The trade-off relationships also appeared in judgment-time task^44^. From the framework of efficient coding the oblique effect may be interpreted as a result of the neural representations of different orientations in sensory systems^48,49^. The experimental phenomena in the presence of reference could be the results of efficient coding in preference-based decisions.

Bayesian inference provides successful description for the phenomena in many cognitive tasks^25,26,29,44,50^. In the paper a Bayesian cue integration models are proposed to describe the experimental results. The process of identification consists of two stages: encoding stage and Bayesian decoding stage. For a given stimulus, the encoding in brain is characterized by the conditional probability distribution of the sensory measurement. The equal precision and variable precision patterns are considered in encoding stage.

In decoding stage, a Bayesian estimator is obtained based on the posterior distribution of the angle $\alpha$ and the cost function. The results show that The Variable-Precision Bayesian models better fit the experimental data and the Variable-Precision Bayesian least squares estimator (BLS_VP) is superior to the others. Our results have revealed that human visual orientation identification can be reasonably described as the outcomes of a Bayesian inference process that optimally integrates the information of known reference into orientation identification. The findings provide an important step toward a better understanding of human visual perception given the known cues.

## Supplementary information

Supplementary Information.

## Data Availability

The experiment code, data, and analysis code are available on https://osf.io/wrxs3/.
