# Supplementary material for: How the known reference weakens the visual oblique effect: a Bayesian account of cognitive improvement by cue influence
Source: Sci Rep. 2020 Nov 20;10:20269. doi: 10.1038/s41598-020-76911-8 (PMC7680155; doi:10.1038/s41598-020-76911-8)
Supplement: Supplementary file 1 — Supplementary Information. [file 41598_2020_76911_MOESM1_ESM.docx]

**Supplementary informations for:**

**How the known reference weakens the visual oblique effect: a Bayesian account of** **cognitive improvement by cue influence**

Renyu Ye^1, 2^· Xinsheng Liu^1*^

^1^ State Key Laboratory of Mechanics and Control of Mechanical Structures, Institute of Nano Science and Department of Mathematics, Nanjing University of Aeronautics and Astronautics, Nanjing, 211106, China.

^2^ School of Mathematics and Physics, Anqing Normal University, Anqing, 246133, China.

***Corresponding author**: Xinsheng Liu

Email: [xsliu@nuaa.edu.cn](mailto:*xsliu@nuaa.edu.cn)

**Appendix: mathematical derivation of equations (4) (5) (6)**

We derive the estimators of Bayesian model in details here. The two different likelihood functions in Equal-Precision and Variable-Precision encoding can be obtained. They are

$p_{EP}(\alpha_{m}|\alpha,\sigma)=\frac{1}{\sqrt{2\pi}\sigma}\text{e}^{\frac{(\alpha_{m}-\alpha)^{2}}{2\sigma^{2}}}$and $p_{VP}(\alpha_{m}|\alpha,w)=\frac{1}{\sqrt{2\pi w^{2}\alpha^{2}}}\text{e}^{\frac{(\alpha_{m}-\alpha)^{2}}{2w^{2}\alpha^{2}}},$ (A1)

respectively, where $\sigma>0$(standard deviation), $w>0$(Weber fraction). There is one free parameter in the encoding stage, $\sigma$or$w$. They are unified as a parameter$\lambda$. The prior distribution of angle is a uniform distribution denoted by Uniform(-23,23). By Bayes’ rule, we can formulate the posterior distributions of the angle $\alpha$ in two encoding patterns as follows

$p_{EP}(\alpha|\alpha_{m},\lambda)=\frac{p(\alpha_{m}|\alpha,\lambda)p(\alpha)}{\int p(\alpha_{m}|\alpha,\lambda)p(\alpha)d\alpha}=\left\{\begin{matrix} \frac{\text{e}^{\frac{(\alpha_{m}-\alpha)^{2}}{2\lambda^{2}}}}{\int_{-23}^{23} \text{e}^{\frac{(\alpha_{m}-\alpha)^{2}}{2\lambda^{2}}}d\alpha}, -23\leq\alpha\leq23 \\ 0 , otherwise \end{matrix}\right.$, (A2)

$p_{VP}(\alpha|\alpha_{m},\lambda)=\frac{p(\alpha_{m}|\alpha,\lambda)p(\alpha)}{\int p(\alpha_{m}|\alpha,\lambda)p(\alpha)d\alpha}=\left\{\begin{matrix} \frac{\text{e}^{\frac{(\alpha_{m}-\alpha)^{2}}{2\lambda^{2}\alpha^{2}}}}{\int_{-23}^{23} \text{e}^{\frac{(\alpha_{m}-\alpha)^{2}}{2\lambda^{2}\alpha^{2}}}d\alpha}, -23\leq\alpha\leq23 \\ 0 , otherwise \end{matrix}\right.$. (A3)

The posterior distribution is a function of the parameter $\lambda$ and sensory measurement $\alpha_{m}$. Consider the two cost functions: $L(\alpha,\alpha_{e})=$ $-\delta(\alpha-\alpha_{e})$ or $L(\alpha,\alpha_{e})=(\alpha-\alpha_{e})^{2}$, where $\delta(\cdot)$ is the Dirac delta function. The Bayesian optimal estimator of angle $\alpha$is the minimum value of the posterior expected cost averaged over all stimuli.

If the cost functions is $L(\alpha,\alpha_{e})=-\delta(\alpha-\alpha_{e})$, the Bayesian estimator is the maximum a posteriori (MAP) estimator as follows:

$\begin{matrix} \theta_{e,MAP}=R+\mathop{argmin}\limits_{\alpha_{e}} \int(-\delta(\alpha-\alpha_{e}))p(\alpha|\alpha_{m},\lambda)d\alpha\\ =R+\mathop{argmax}\limits_{\alpha_{e}} \int\delta(\alpha-\alpha_{e})p(\alpha|\alpha_{m},\lambda)d\alpha\\ =R+\mathop{argmax}\limits_{\alpha_{e}} \{p(\alpha_{e}|\alpha_{m},\lambda)\cdot\mathrm{I}_{(-23,23)}(\alpha_{e})\} \end{matrix}$ (A4)

where $I(\cdot)$ is an indicator function. By substituting these posterior distributions (A2) and (A3) into equations (A4), we obtain the corresponding maximum a posteriori estimators under the Equal-Precision and Variable-Precision, respectively. That is, the equations (4) and (5) hold.

$\theta_{e,MAP-EP}=\left\{\begin{matrix} R-23, \alpha<-23 \\ R+\alpha_{m}, -23\leq\alpha\leq23 \\ R+23, \alpha> 23 \end{matrix}\right..$ (A5)

$\begin{matrix} \theta_{e,MAP-VP}=R+\mathop{argmax}\limits_{\alpha_{e}} \{\text{e}^{\frac{(\alpha_{m}-\alpha)^{2}}{2\lambda^{2}\alpha^{2}}}\cdot\mathrm{I}_{[-23,23]}(\alpha_{e})\} \\ =\left\{\begin{matrix} R-23, \alpha< -23 \\ R+\alpha_{m}[\frac{-1+\sqrt{1+4\lambda^{2}}}{2\lambda^{2}}], -23\leq\alpha\leq23 \\ R+23, \alpha>23 \end{matrix}\right.. \end{matrix}$ (A6)

The $\theta_{e,MAP-VP}$in equation (A6) is obtained by setting the derivative on $\lambda$ to zero.

If the cost function is $L(\alpha,\alpha_{e})=(\alpha-\alpha_{e})^{2}$, the equation (2) can be written as

$\theta_{e}=R+\alpha_{e}=R+\mathop{argmin}\limits_{\alpha_{e}} \int(\alpha-\alpha_{e})^{2}p(\alpha|\alpha_{m},\lambda)d\alpha.$ (A7)

Let $G(\alpha_{e})=\int(\alpha-\alpha_{e})^{2}p(\alpha|\alpha_{m},\lambda)d\alpha,$ then $\frac{dG(\alpha_{e})}{d\alpha_{e}}=0$implies that

$2\alpha_{e}-2\int\alpha p(\alpha|\alpha_{m},\lambda)d\alpha=0.$ (A8)

From Eq. (A8), the equation (6) holds. The Bayesian estimator is the mean of the posteriori distribution, referred to as the Bayesian least squares estimator (BLS).

**Preliminary experiment of orientation identification**

The preliminary experiment investigated the effect of a known oriented reference line on global orientation identification. The apparatus and procedure of the preliminary experiment are same as the experiment in manuscript. The stimulus orientations were randomly drawn from a discrete uniform distribution with 7 values (0°, 15°, 30°, 45°, 60°, 75°, and 90°). Each orientation was repeated 20 times, for a total of 140 trials. Then the experimental sample stimuli set was established. Because the oblique orientations near the 45-degree reference line may be least affected by the cardinal direction, we chose the orientation of reference line at 45 degrees. Before the experiment trials, each subject performed 100 trials with the correct feedback (Fig.1a in manuscript). The stimulus orientations of practice trials were drawn randomly from between 0° and 90°, and rounded to the nearest integer.

As expected, in the absence of reference, the standard deviations (*SD*) of estimated orientations are lower for the cardinal orientations (i.e., 0 and 90 degrees), while the standard deviations (*SD*) are higher for the oblique orientations (i.e., 15, 30, 45, 60 and 75 degrees) (Table S1, Fig. S1). The oblique effect occurs, which is similar to the results of previous study by Tschopp-Junker et al. (2010) (ref.37 in the manuscript) In the presence of reference line, the *SD* and *BIAS* of estimates are obviously different from those without reference (make 2(situation) × 7(orientation) repeated-measures two-way ANOVA, *SD*: $F_{1\text{，42}}=103.84, p<0.0001$, *BIAS*: $F_{1\text{，42}}=5.54, p=0.023$). The oblique effect is weakened due to the improvement of the precision of the oblique orientations, especially in the vicinity of the reference line, but the oblique effect is not completely suppressed (Fig. S1). The identification precision of orientations in the vicinity of the cardinal orientations is no significant difference in two situations (Table S2). In each situation, there are no significant differences among subjects in terms of *BIAS* and *SD* values (Table S3). Experimental results show that the known reference line significantly influence the orientation identification in the vicinity of the reference line. As the target orientation moves toward the reference line, the impact becomes greater.

**Supplementary Figures**

**
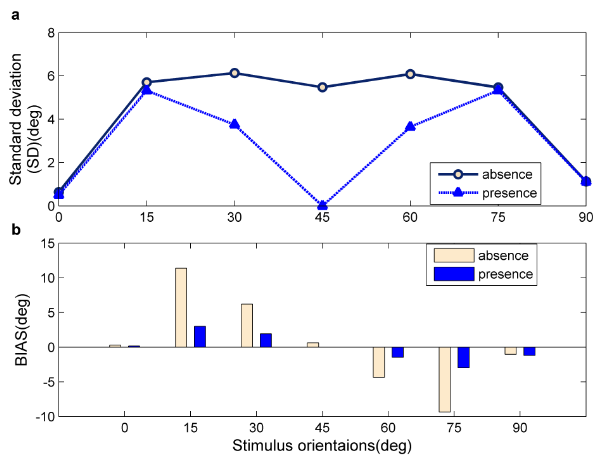
**

**Figure S1.** Orientation-identification results of preliminary experiment across four subjects. (**a).** The standard deviations (*SD*) across four subjects as a function of stimulus orientations. The triangles represent the *SD* values in presence of reference and the dots represent the ones without reference. Each data point is the average of estimates of the same orientation for four subjects in each situation. (**b).** The contrast histogram of orientation identification’s *BIAS* across four subjects in the two situations.


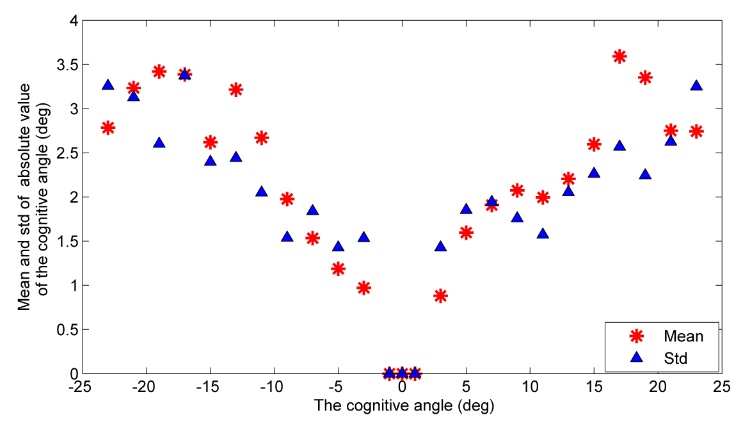


**Figure S2. Scatter plot of mean and standard deviation of the cognitive angles over 11 subjects.** Red stars indicate the mean of absolute value of the cognitive angles. Blue triangles indicate the standard deviation of absolute value of the cognitive angles. The scatter plot shows that the correlation between mean and standard deviation of absolute value of the cognitive angles.

Based on our experimental data, we calculated the correlation coefficient between the mean and the standard deviation of the absolute value of the cognitive angle and we obtain the correlation coefficient r= 0.9046.

**Supplementary Tables**

| **Table S1** The experimental results without the reference in preliminary experiment | | | | | | |
| --- | --- | --- | --- | --- | --- | --- |
|  | *BIAS* | | *SD* | | *MSE* | |
| Reference | absence | presence | absence | presence | absence | presence |
| **0** | 0.275 | 0.175 | 0.64 | 0.501 | **0.485** | 0.281 |
| **15** | 11.375 | 3.002 | 5.705 | 5.325 | **162.939** | 37.359 |
| **30** | 6.201 | 1.925 | 6.132 | 3.751 | **76.04** | 17.777 |
| **45** | 0.625 | 0 | 5.476 | 0 | **30.375** | 0 |
| **60** | -4.35 | -1.475 | 6.083 | 3.644 | **55.925** | 15.457 |
| **75** | -9.35 | -2.975 | 5.466 | 5.328 | **117.295** | 37.235 |
| **90** | -1.05 | -1.2 | 1.132 | 1.114 | **2.385** | 2.681 |

**Table S2** The results of one-way ANOVA for the precision of each stimulus

in preliminary experiment

| Sample orientation | 0 | 15 | 30 | 45 | 60 | 75 | 90 |
| --- | --- | --- | --- | --- | --- | --- | --- |
| *F*(1,6) | 0.93 | 1.52 | 25.52 | 309.22 | 83.17 | 0.07 | 2.65 |
| *P* value | 0.3731 | 0.2637 | 0.0023 | 2.17E-06 | 9.77E-05 | 0.806 | 0.1548 |

**Table S3** The results of ANOVA for inter-subject difference in bias and precision in preliminary experiment (4 subjects*7 orientations)

| Reference situation | Absence | Presence | Absence | Presence |
| --- | --- | --- | --- | --- |
| metrics | bias | bias | precision | precision |
| *F*(3,18) | 1.4 | 0.26 | 0.3 | 1.71 |
| *P* value | 0.274 | 0.8527 | 0.8227 | 0.2 |

**
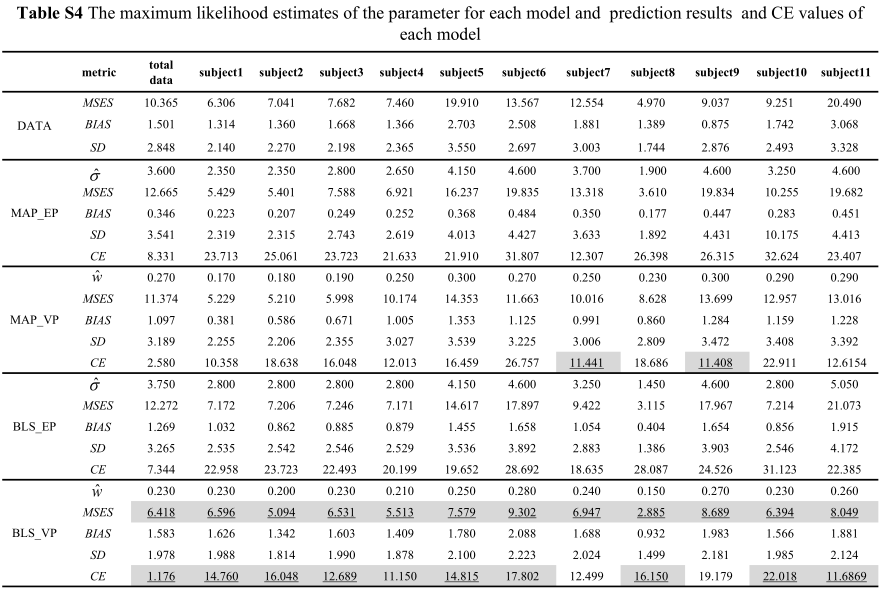
**
